# Abnormal Social Reward Responses in Anorexia Nervosa: An fMRI Study

**DOI:** 10.1371/journal.pone.0133539

**Published:** 2015-07-21

**Authors:** Esther Via, Carles Soriano-Mas, Isabel Sánchez, Laura Forcano, Ben J. Harrison, Christopher G. Davey, Jesús Pujol, Ignacio Martínez-Zalacaín, José M. Menchón, Fernando Fernández-Aranda, Narcís Cardoner

**Affiliations:** 1 Bellvitge University Hospital - Institut d'Investigació Biomèdica de Bellvitge (IDIBELL), Barcelona, Spain; 2 Department of Clinical Sciences, School of Medicine, University of Barcelona, Barcelona, Spain; 3 Melbourne Neuropsychiatry Centre, The Department of Psychiatry, The University of Melbourne, Melbourne, Australia; 4 CIBER Salud Mental (CIBERSAM), Instituto de Salud Carlos III, Barcelona, Spain; 5 Department of Psychobiology and Methodology of Health Sciences, Universitat Autònoma de Barcelona, Spain; 6 IMIM Research Institute at the Hospital de Mar, clinical research group in human pharmacology and neuroscience, Barcelona, Spain; 7 CIBER Fisiopatología Obesidad y Nutrición (CIBERObn), Instituto de Salud Carlos III, Barcelona, Spain; 8 Orygen, The National Centre of Excellence in Youth Mental Health, Melbourne, Australia; 9 MRI Research Unit, Hospital del Mar, CIBERSAM G21, Barcelona, Spain; University of Manchester, UNITED KINGDOM

## Abstract

Patients with anorexia nervosa (AN) display impaired social interactions, implicated in the development and prognosis of the disorder. Importantly, social behavior is modulated by reward-based processes, and dysfunctional at-brain-level reward responses have been involved in AN neurobiological models. However, no prior evidence exists of whether these neural alterations would be equally present in social contexts. In this study, we conducted a cross-sectional social-judgment functional magnetic resonance imaging (fMRI) study of 20 restrictive-subtype AN patients and 20 matched healthy controls. Brain activity during acceptance and rejection was investigated and correlated with severity measures (Eating Disorder Inventory -EDI-2) and with personality traits of interest known to modulate social behavior (The Sensitivity to Punishment and Sensitivity to Reward Questionnaire). Patients showed hypoactivation of the dorsomedial prefrontal cortex (DMPFC) during social acceptance and hyperactivation of visual areas during social rejection. Ventral striatum activation during rejection was positively correlated in patients with clinical severity scores. During acceptance, activation of the frontal opercula-anterior insula and dorsomedial/dorsolateral prefrontal cortices was differentially associated with reward sensitivity between groups. These results suggest an abnormal motivational drive for social stimuli, and involve overlapping social cognition and reward systems leading to a disruption of adaptive responses in the processing of social reward. The specific association of reward-related regions with clinical and psychometric measures suggests the putative involvement of reward structures in the maintenance of pathological behaviors in AN.

## Introduction

Anorexia nervosa (AN) is a severe and disabling psychiatric disorder. With limited evidence-based treatments available, at least 25% of patients show poor clinical outcome and high levels of functional and social impairment [1–5]. These data highlight the need for a better understanding of the underlying pathophysiological bases of AN, including the identification and precise delineation of the complex neural systems involved [6]. Current theoretical models describe AN as a multifactorial disorder [7,8] and, social factors, including both the impact of social environment and how individuals interact and process social information, are considered highly relevant to the development, maintenance and prognosis of the disorder [1,9]. Indeed, AN patients generally struggle to maintain interpersonal social relationships, with evidence of social difficulties and social anxiety symptoms, both in the premorbid state and after the disorder’s onset [10,11]. However, little is known about the neural substrates responsible of these abnormal responses to social stimuli or their relevance in the disorder.

Reward-based processes have been highlighted as powerful and natural modulators of social interactions [12,13]. Indeed, social information is acquired using the same mechanisms of basic reward-based learning, (e.g. reward evaluation and associative learning) [12], such that past social experiences are used to predict future social outcomes, attempting to maximize rewards and avoid punishments [14]. At the neural level, reward- and punishment-based learning involves midbrain dopaminergic neurons sending large-scale projections to the ventral striatum, the amygdala, the ventromedial prefrontal cortex, the orbitofrontal and frontal opercula-insular cortices [12,15–19]. All these regions have been involved in reward response prediction, either to primary (e.g. the taste of food) or more complex reinforcements such social stimuli [14]. For example, the reward system has been shown to respond to gaze direction, images of romantic partners and even to the experience of being liked, among others [14,20]. Direct comparisons between social and other stimuli have also shown the overlapping nature of reward system responses to a broad variety of rewards [19,21]. Given the complex nature of social relationships, which require the integrated participation of a number of functions (e.g. social cognition, emotion processing and regulation [13,22]), these tasks have also shown to activate the reward system in conjunction with other areas, for example those involved in theory of mind and self-related regions [20].

In AN, increasing evidence has suggested altered responses of this so-called brain reward system. Early studies suggested a rewarding effect of starvation itself through an hypercortisolemic and hyperdopaminergic state [23], and, in the same line, the animal model of self-starvation/activity-based anorexia (ABA) has implicated imbalances in the brain reward system in AN, driven merely by modifications in food consumption and starvation [24]. Further development of conditioned processes based on this aberrant reward-system response have been also implicated in the pathophysiology of AN, where primary rewarding stimuli (such as food) might become aversive, and negative stimuli might become rewarding, as suggested by the *contamination reward theory* [25,26]. Biological evidences of this imbalance have come mainly from alterations in the concentrations of dopamine and its D2 receptor found both in AN patients and recovered subjects [27,28], as well as from functional magnetic resonance (fMRI) studies, which have shown abnormal responses of regions such as the ventral striatum, the anterior insula and the ventromedial prefrontal cortex [27,29,30]. For example, the ventral striatum has been found to present either a dysfunctional hyperactivation to the visualization of underweight bodies [31], an exaggerated [32,33] or a decreased [34] response to pleasant/sweet tastes, and even found to be non-discriminative between wins and losses in a monetary reward task [35]. These findings have been proposed as a potential trait marker of the disorder, given the presence of abnormal responses to disorder-specific and disorder-nonspecific stimuli [29] in both ill and recovered AN patients [33]. In the context of social stimuli, AN patients might present similar alterations in their responses to reward. Data from behavioral studies have suggested a negative bias in social relationships: patients with AN perceive low reward from- and are avoidant of- social contexts and are oversensitive and attention-biased towards rejection [1,36–40]. These behavioral responses are modulated by the so-called approaching/avoidance systems [41,42], which in AN might be affected though alterations in personality traits linked to these systems [43]. Specifically, AN present consistent heightened scores in *sensitivity to punishment* and putative alterations in *sensitivity to reward*, thought to be vulnerability factors inherently associated with the illness [43–46]. Taken all together, the question arises as to whether altered brain reward responses are implicated in the processing of social stimuli in AN, and if present, whether they involve the same areas found to be altered for non-social rewards or expand to an extended network. Likewise, there are scarce evidences regarding the level to which sensitivity to reward and punishment might be modulating the responses to social stimuli.

We therefore investigated brain responses to social reward (acceptance) and punishment (rejection) in patients with restrictive-subtype AN in an fMRI experiment. Specifically, we used a modified version of a peer-oriented social judgment paradigm [20,47], previously shown to activate reward- and social processing- related brain regions, including the ventral striatum, the insular cortex and dorsal and ventromedial prefrontal cortices. We hypothesized that AN patients, when receiving socially rewarding peer feedback, would demonstrate reduced activity in these regions. When they received negative feedback we considered two possible outcomes. Considering AN heightened sensitivity and attention-bias to punishment and social rejection, one possibility would be to detect increased activation of regions engaged in attentional processing or in social rejection (e.g. the dorsal anterior cingulate and anterior insula cortices [48]). Alternatively, we might find evidences for a primary dysfunctional enhancement of reward-related activity, as has been found for other non-naturally rewarding stimuli in AN [31]. We also anticipated an interaction between reward brain areas and sensitivity to reward and punishment and explored whether an abnormal brain response to social reward/punishment would be modulated by the severity of the disorder.

## Material and Methods

### Participants

Twenty female patients with Anorexia Nervosa, restricting subtype [49] (mean age 28.40 years; SD 9.30 years) were recruited consecutively from admissions at the day patient program, Eating Disorders Unit of Bellvitge University Hospital, Barcelona between 2011 and 2012. Diagnoses were conducted by experienced psychologists/psychiatrists (E.V., I.S., F.F-A.) following DSM-IV TR criteria and using a semi-structured clinical interview (Structured Clinical Interview for DSM-IV Axis I Disorders) [50]. Five patients (25% of the sample) were on pharmacological treatment, as described elsewhere ([51]; Table 1). Comorbid psychiatric disorders—including any other eating disorder-, any neurological condition and abuse of any substance with the exception of nicotine were exclusion criteria. None of the patients met criteria for hospital admission at the time of scanning on the basis of physical consequences of excessive starvation.

**Table 1:** Demographic and clinical description of the subjects included in the sample.

		AN patients (n = 20)	Healthy controls (n = 20)	Between group differences		
				t Statistic	p	Cohen's d
Age: mean in years (sd), range		28.40 (9.30), 18–49	28.15 (8.62), 19–52	0.09	.93	0.03
Handedness: right/left (number of subjects)		19/1	19/1	-	-	-
Educational level: mean in years of studies (sd), range		15.85 (3.56), 12–23	16.45 (2.46), 10–21	0.62	.54	0.19
Age at the onset: mean in years (sd), range		21.30 (9.26), 11–48	-	-	-	-
Illness duration: mean in months (sd), range		85.20 (76.88), 12–240	-	-	-	-
BMI: mean (sd), range*		16.94 (1.26), 14–18	20.99 (1.82), 18–25	8.47	< .001	2.59
EDI-2: mean (sd), range		66.79 (44.28), 13–178	13.53 (7.37), 3–28	5.17	< .001	1.68
	Drive for Thinness	10.35 (7.34), 0–21	0.90 (1.55), 0–6			
	Bulimia	1.25 (1.59), 0–4	0.05 (0.22), 0–1			
	Body dissatisfaction	11.20 (8.67), 0–27	2.40 (2.78), 0–9			
	Ineffectiveness	7.80 (7.95), 0–28	0.75 (1.16), 0–3			
	Perfectionism	7.10 (4.87), 1–17	3.60 (3.57), 0–12			
	Interpersonal distrust	3.40 (3.72), 0–11	0.65 (1.35), 0–5			
	Interoceptive awareness	6.45 (6.16), 0–20	0.25 (0.34), 0–2			
	Maturity fears	5.35 (4.84), 0–16	2.70 (2.89), 0–11			
	Asceticism	5.65 (3.96), 1–16	1.25 (1.12), 0–4			
	Impulse regulation	3.30 (4.52), 0–14	0.25 (0.64), 0–2			
	Social insecurity	4.95 (4.81), 0–17	0.40 (1.00), 0–4			
SPSRQ total: mean (sd), range		21.35 (7.06), 8–34	15.95 (6.95), 6–30			
SPSRQ Subscales:						
	SP	12.85 (5.45), 2–20	8.20 (4.37), 2–17	2.98	= .005	0.94
	SR	8.50 (4.22), 2–15	7.75 (4.35), 2–16	0.55	= .58	0.18
LSAS: mean (sd), range		44.60 (26.53), 9–89	25.65(16.60), 3–67	2.70	= .011	0.86
HDRS: mean (sd), range		3.30 (2.94), 0–10	0.90 (1.07), 0–3	3.43	= .002	1.08
HARS: mean (sd), range		4.95 (5.79), 0–22	1.65 (1.39), 0–4	2.48	= .025	0.78
Pharmacological treatment (n):						
	Selective serotonin reuptake inhibitors	3	-			
	Tricyclic antidepressant	1	-			
	Sedative antipsychotic +tricyclic antidepressant	1	-			

BMI: Body mass index. EDI-2: Eating Disorders Inventory-2. LSAS: Liebowitz Social Anxiety Scale. HDRS: Hamilton Depression Rating Scale. HARS: Hamilton Anxiety Rating Scale.

* Patients received at least one week of supervised meals and hydration before the MRI, and were scanned in the afternoon, 2–4 hours after lunch.

20 healthy controls (20 females, mean age 28.15, SD 8.62) were recruited from the same sociodemographic area and matched by gender, mean age, handedness and mean educational level with the patients (Table 1). Controls were screened in order to exclude any psychiatric or other medical condition by means of the General Health Questionnaire (GHC-28, [52]) and a clinical semi-structured interview [53]. None of the controls presented subthreshold symptoms for any eating disorder and their body mass index (BMI) was within the normal range.

### Ethics statement

The ethical committee of clinical research (CEIC) of the Bellvitge University Hospital approved the study protocol, which was in compliance with the national legislation and the principles expressed in the Declaration of Helsinki. All participants gave written informed consent after detailed description of the study.

### Clinical measures

For all participants, severity of symptoms and psychological features involved in eating disorders were assessed with the self-reported Eating Disorder Invertory-2 (EDI-2) scale [54]. The Sensitivity to Punishment and Sensitivity to Reward Questionnaire (SPSRQ) [55] and the Liebowitz Social Anxiety Scale (LSAS) [56] were also collected. Additionally, measurements of depressive and anxiety symptoms were collected by means of the Hamilton Depression Rating Scale (HDRS [57]) and the Hamilton Anxiety Rating Scale (HARS [58]).

### Social Judgment Task

A modification of the task originally reported in Davey et al.[20] was used in the current study. Participants were assessed on two different days, approximately five days apart. On the first day, participants were asked to participate in a multi-center study about the influence of first impressions on deciding whether or not people would like to meet someone. They were presented a face database containing 70 people's faces with neutral expression (35 male and 35 female faces)—supposed to be study’s participants from other collaborating centers- and were asked to decide if they would like to meet them or not (*acceptance/rejection*), rating their decision in a 10-point Likert-type scale—10 being the maximum for liking to meet someone (*score pre-scanning*). Likewise, participants had a photograph taken, which was supposedly sent and reciprocally scored by the database participants. This feedback was given during the fMRI scanning on the second day of assessment. In reality, the face database integrated pictures selected from a larger pre-existing and public available face database [59], and at the end of the experiment, participants were debriefed about the deception involved.

**Fig 1:**
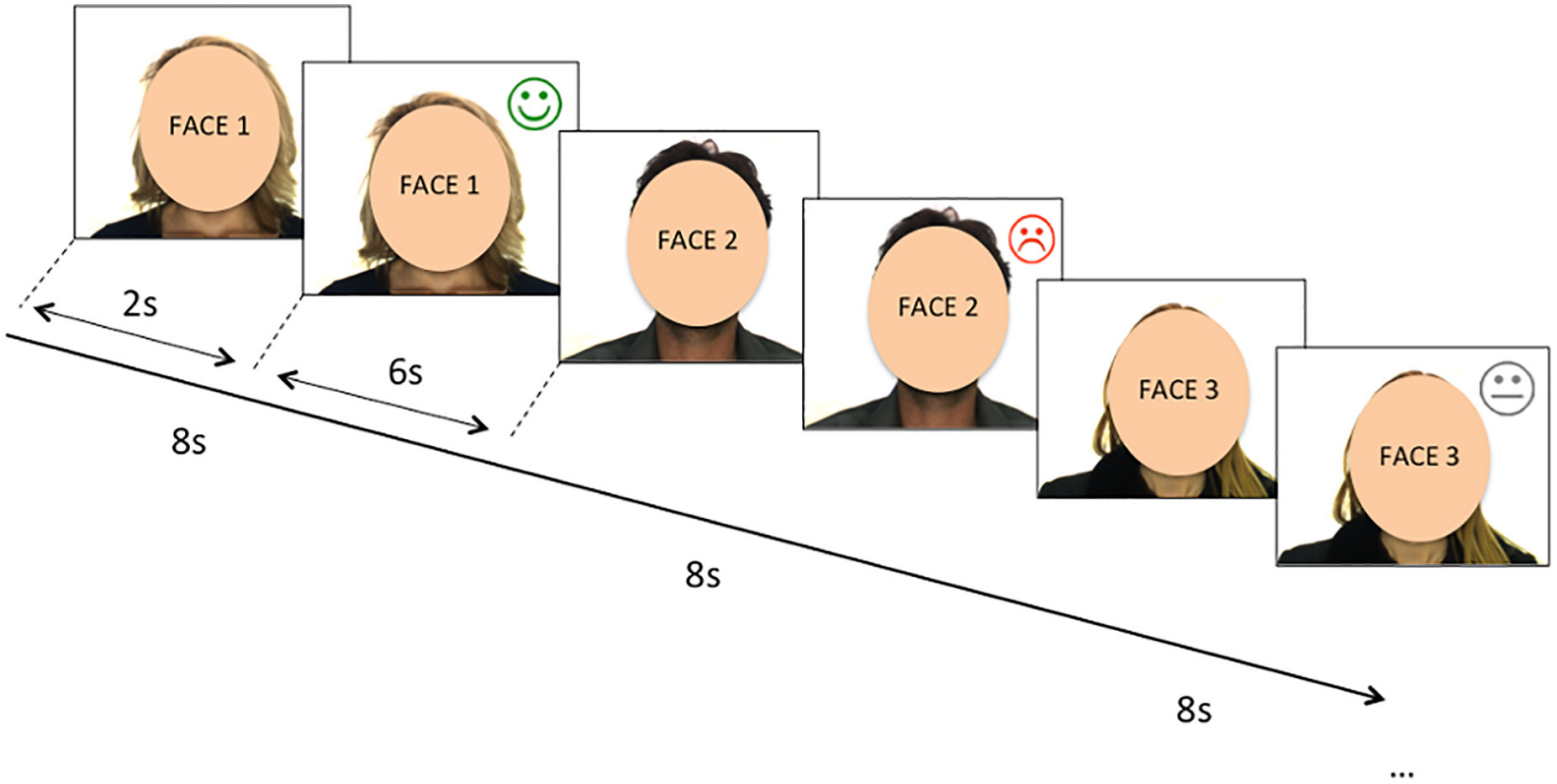
Diagram of the Social Judgment Task used in the fMRI session. Participants received social feedback based on the willingness to be met by other participants. Each facial stimulus (represented in by ovals instead of the originally presented faces) was presented for a total of 8 second-blocks, with an overlapping feedback symbol during the last 6 seconds. Acceptance, rejection or no-feedback (control condition) was indicated by a happy, sad, or neutral draw of a face. Originally presented images were contained in a preexisting face database: Martinez AM, Benavente R. The AR Face Database CVC Tech. Report #24 [Internet]. 1998. Available: http://www2.ece.ohio-state.edu/~aleix/ARdatabase.html.

During the fMRI scanning, participants viewed a total of 54 of the 70 rated on the first assessment day. Each picture was presented for 8 seconds, and during the last 6 seconds a feedback symbol (a happy, sad or neutral draw of a face) was additionally displayed on the top right corner of the picture (Fig 1). Participants were instructed that happy face symbols indicated acceptance, and sad faces rejection. Neutral faces appeared when people supposedly could not be contacted to give feedback, which formed the control condition of the experiment. The 54 presented faces and the feedback responses were pseudo-randomly determined to ensure good balance between gender (27 male, 27 female) and between the three conditions (17 acceptance responses, 18 rejection responses and 19 control condition responses). The paradigm was presented visually on a laptop computer running E-Prime software on Windows (Psychology Software Tools, Inc., Sharpsburg, PA, USA, www.pstnet.com). Magnetic resonance imaging-compatible high-resolution goggles were used to display the stimuli.

After the scanning session, participants were presented again with the complete face database (70 faces). For each face, they were asked to recall if it appeared during the scanning and in each case what type of feedback the person had given. Participants were also asked about the first impression they had of each face on the first day (10-point Likert-type scale, *score post-scanning*). This assessment allowed exploration of potential attention and memory biases. A visual analogue scale was used to evaluate how they felt after receiving each one of the three types of feedback (scores ranging from 0 to 10). Finally, after debriefing about the nature of the study, participants were asked to rate how much they believed they had received true feedback (scores ranging from 0 to 10).

### Behavioral measures

Accuracy of recall on which faces had been displayed during the MRI session was compared between groups using a two-sample *t*-test. Next, the number of correctly remembered feedback responses was compared across groups and conditions by means of a mixed-design ANOVA analysis: task condition (acceptance, rejection, neutral) was included as the within-group variable and group (controls, patients) as the between-group variable (3x2 mixed ANOVA). Then, to compare, between groups, score changes across the two time points (pre-and post- scanning) and the three conditions, a second ANOVA analysis was conducted, with task condition and pre-post scores as the within-group variables, and group as the between-group factor (3x2x2 mixed ANOVA). Additionally, a similar 3x2 ANOVA was conducted to compare, between-groups and conditions, how the subjects felt when receiving each type of feedback. Finally, a two-sample t-test was conducted to compare, between-groups, how much they believed they were being truly evaluated. Behavioral analyses were performed in Statistical Package for the Social Sciences (SPSS) v20 on a Windows platform. Level of significance was set at p<0.05.

### Imaging acquisition and preprocessing

A 1.5-T Signa Excite system (General Electric Milwaukee, WI, USA) magnetic resonance, equipped with an 8-channel phased-array head coil and single-shot echoplanar imaging software was used. The functional sequence consisted of gradient recalled acquisition in the steady state (repetition time (TR) = 2000 ms, echo time (TE) = 50ms and pulse angle, 90°) in a 24 cm field of view, 64 x 64 pixel matrix and a slice thickness of 4mm (inter-slice gap, 1.5 mm). A total of 22 interleaved sections, parallel to the anterior—posterior commissure line, were acquired to generate 216 whole-brain volumes, excluding four initial dummy volumes to allow the magnetization to reach equilibrium.

Data were processed on a Macintosh platform running Matlab version 7.14 (The MathWorks, Inc) and statistical parametric mapping software version 8 (SPM8). Within participants, time-series of acquired images were initially realigned to the mean image by using a least squares and a 6-parameter (rigid body) spatial transformation. Images were then normalized to the standard echoplanar imaging (EPI) template in SPM and resliced in Montreal Neurological Institute (MNI) space (resulting voxel size 2 mm^3^). Finally, they were smoothed with an 8 mm isotropic Gaussian filter. All image sequences were routinely inspected for potential movement or normalization artifacts.

### Imaging processing and imaging analyses

For each participant, the onset and offset timing of the conditions (each 6-second block of the acceptance, rejection and neutral conditions, as well as the first 2-seconds of no-feedback), was convolved with a canonical hemodynamic response function to model the acquired BOLD signal. A high-pass filter was used to remove low-frequency noise (cut off period = 1/128 Hz). At a first single-subject level of analysis, contrasts were defined as: i) the acceptance condition *minus* the control condition; and ii) the rejection condition *minus* the control condition. Within-group contrast images were then carried to a mixed effects second-level, creating two-sample t-tests at each voxel to compare between-group brain activations.

Statistical analyses at the second level involved a combination of voxel and cluster correction methods providing a significance level equivalent to a Family Wise Error corrected p (pFWE)<0.05. Specifically, individual voxel threshold was set at an uncorrected p<0.005, while minimum spatial cluster extent (min. K_E_) required to satisfy a pFWE<0.05 was determined by 1000 Monte Carlo simulations using the Alphasim algorithm as implemented in the SPM RESting-state fMRI data analysis Toolkit (REST) toolbox in Matlab [60]. Other input parameters included a connection radius of 5 mm and the actual smoothing value of each statistical comparison (between 13 and 16mm). Cluster extents were determined using a whole-brain mask for within-group activations and single masks containing combined (both groups) brain activations for the between-group comparisons (whole brain mask: 337,701 voxels, min. K_E_ = 124 for acceptance, min K_E_ = 147 for rejection; masks with combined brain activations: 4,568–27,851 voxels; min. K_E_ ranged between 29–99).

Additional analyses were conducted to explore the relationship between clinical measurements and brain activations during task performance. Firstly, to assess for potential and differential associations between sensitivity to reward/punishment and brain activations between groups, SPSRQ scores were included in two separate between-group interaction analyses (sensitivity to reward during acceptance and sensitivity to punishment during rejection). Secondly, brain activations in patients were correlated with EDI-2 scores in two separate regression analyses (acceptance, rejection). Levels of significance were set based on the same cluster correction methods used for the main analyses. For the interaction SPSRQ analyses, masks contained the combined activation of patients and controls in both the acceptance and rejection, while separate masks containing patients' activations during the acceptance and the rejection conditions were used for the correlation analyses with EDI-2 (masks contained between 4,787–36,673 voxels; min. K_E_ = 4–118). Age and depressive symptoms (see below) were included as nuisance covariates in all the analyses.

## Results

### Clinical and demographic variables

There were no statistically significant differences in age, handedness or educational level between patients and controls. As expected, body mass index (BMI) and EDI-2 measurements were significantly different in patients and controls, with lower mean BMI and higher mean EDI-2 scores in patients.

SPSRQ subtest scores indicated higher sensitivity to punishment in patients, with no differences in sensitivity to reward. Higher LSAS scores were also found in patients although differences did not survive Bonferroni correction for multiple testing. Similarly, depressive and anxiety symptoms were higher in patients compared to controls, but Bonferroni-corrected statistical significance was only observed for depressive symptoms (Table 1). Since anxiety and depressive symptoms were highly correlated (r = .86, p< .001), we only included depressive symptoms as a nuisance covariate in our analyses.

### Behavioral measures

Both groups remembered with high accuracy which faces appeared during the fMRI task (AN patients: 69%, Controls: 65%). There were no interaction effects or between-group differences in the accuracy of recall to the different types of feedback; however, across conditions, all participants more accurately remembered being rejected in comparison to being accepted (p <.001) or receiving no feedback (p <.001; condition effect: F(2,76) = 16.54, p <.001). Similarly, there were no interaction effects or between-group differences in the *pre* and *post-scanning scores* across conditions, and all participants gave both higher *pre* and *post-scanning* ratings to faces that provided rejection feedback compared to acceptance or no feedback (both p <.001; condition effect: *F*(2,76) = 36.43, p <.001).

There were no interaction effects or between-group differences on how participants felt after receiving any type of feedback, and all of them liked more being accepted than rejected (p <.001) or receiving no feedback (p <.001; condition effect: F(2,74) = 83.32, p <.001). All participants indicated that they believed the participant ratings were genuine (mean (SD) out of 10: AN patients: 9.4 (1.30); Controls: 9.12 (1.21)). The results are summarized in S1 Table.

### Imaging results

#### Main analyses: within-group results

In response to acceptance feedback, both groups showed an overlapping activation of the dorsal and ventral medial prefrontal cortices. Controls presented an additional activation of the ventral striatum and bilateral anterior insular cortices, whereas patients showed an additional activation of an area including the parahippocampal gyrus, hippocampus and amygdala.

**Fig 2:**
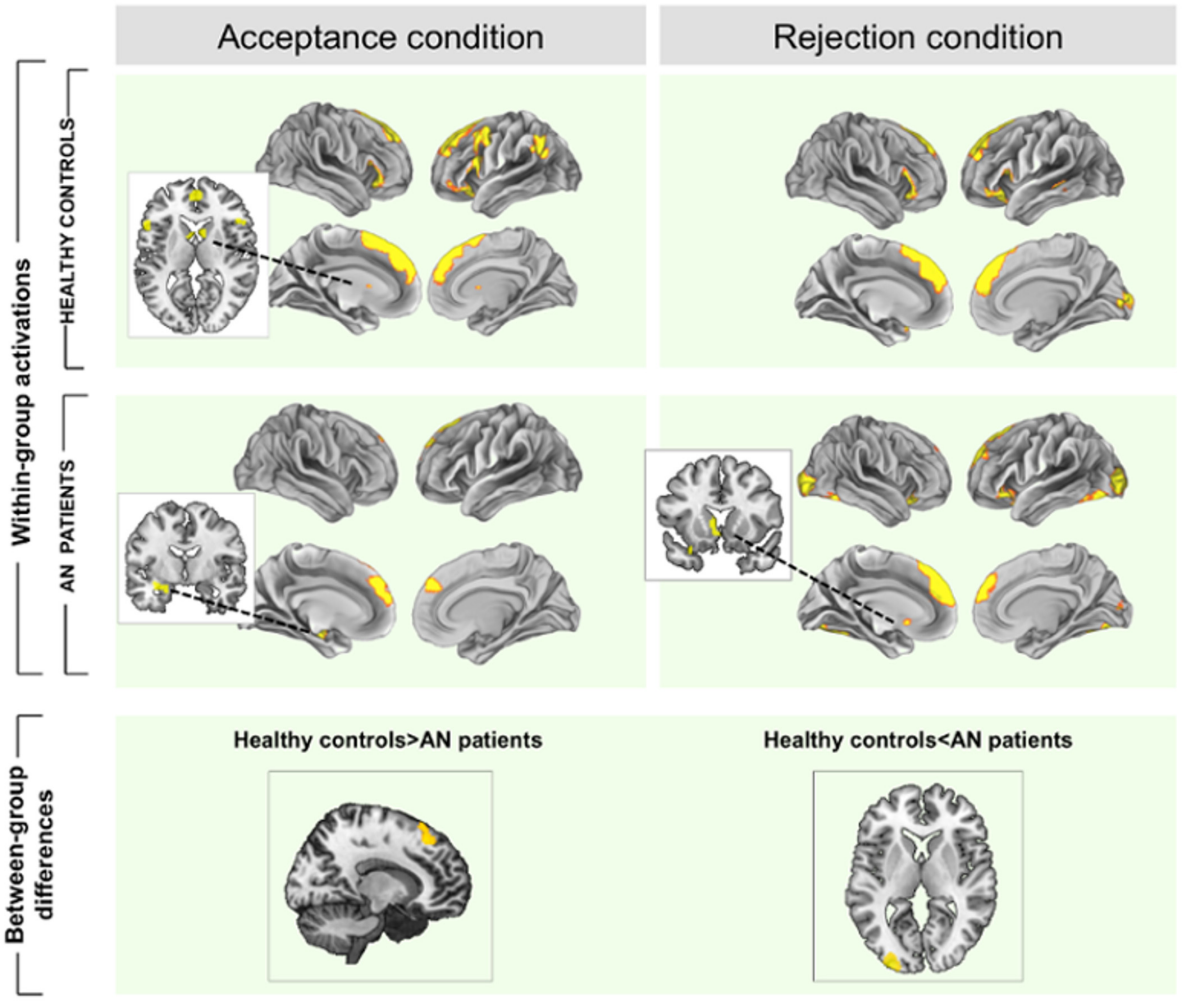
Within and between-group brain activations during acceptance and rejection feedback. Brain hyperactivations (i.e. contrast acceptance/rejection>control condition) are depicted in yellow and deactivations (i.e. contrast acceptance/rejection<control condition) are in blue. A and B represent within-group activations in A = controls and B = patients. Below, results for the comparison controls>AN patients and for the comparison AN patients>controls. Color bars represents T value, only for between-group comparisons. Images are displayed in neurological convention (left is left).

Conversely, both groups presented a similar pattern of brain activation in response to rejection, with enhancement of the dorsomedial prefrontal cortex, anterior insular cortices and primary and secondary visual areas. Patients showed an additional activation of the ventral striatum, specifically in the ventral part of the caudate nucleus (Table 2, Fig 2).

**Table 2:** Within and between-group activations of extended brain regions during the performance of the task.

	Healthy controls						AN patients						Group comparisons					
		Anatomy ^1^			Stats ^2^			Anatomy ^1^			Stats ^2^			Anatomy ^1^			Stats ^2^	
		x	y	z	K_E_	Z		x	y	z	K_E_	Z		x	y	z	K_E_	Z
**Acceptance > neutral contrast**													**Healthy controls> AN patients**					
	Medial superior frontal cortex (BA 8 and BA9, extending to BA6 and BA10)	-14	48	38	5 133	4.95	Medial superior frontal cortex (BA 8 and BA9, extending to BA10)	-10	36	60	1 104	3.92	Medial superior frontal cortex (BA8, extended to BA9)	10	32	50	127	3.38
		10	28	62		4.56		6	48	34		3.34		10	28	62		2.87
		-14	36	56		4.39		-8	54	34		3.30						
	Left dorsolateral prefrontal cortex/left anterior insular cortex	-48	14	24	2 000	4.08	Left parahippocampus, extended to hippocampus, fusiform and amygdala cortices	-34	-12	-22	330	3.75						
		-40	2	40		3.82		-30	-14	-14		3.46						
		-44	2	50		3.71		-38	-20	-26		3.45						
	Left temporo-parietal junction	-56	-56	46	696	4.01												
		-50	-60	52		3.42							**Healthy controls<AN patients**					
		-52	-58	30		3.37							No areas					
	Right frontal operculum/right anterior insular cortex	46	32	-8	462	3.95												
		52	26	-2		3.85												
		46	26	-14		3.68												
	Bilateral ventral striatum (caudate)	8	10	10	125	3.45												
		-6	8	8		3.21												
**Rejection > neutral contrast**	Medial superior frontal cortex	-8	44	52	3 580	5.34	Medial superior frontal cortex	-4	52	22	2 302	4.77	**Healthy controls> AN patients**					
		-8	50	44		5.07		-8	38	58		4.54	No areas					
		12	42	50		4.59		-12	54	32		3.94						
	Left inferior frontal cortex, triangular and orbital parts/Left anterior insular cortex.	-50	18	6	680	4.12	Right inferior frontal gyrus, operculum/right anterior insular cortex	32	24	-18	250	3.91						
		-38	26	-16		3.45	Left inferior frontal gyrus, operculum/ Left anterior insular cortex.	-30	16	-24	276	3.26						
		-44	36	-16		3.32		-28	22	-10		3.01						
	Right inferior frontal cortex, triangular and opercular parts/right anterior insular cortex	52	28	-6	292	4.00		-48	30	-14		3.00						
		60	24	20		2.82	Left ventral striatum (caudate)	-8	6	4	155	2.97	**Healthy controls<AN patients**					
		52	24	10		2.81		-6	14	-6		2.90	Visual cortex (BA18)	-30	-98	6	262	3,79
	Left middle temporal cortex	-50	-42	-2	226	3.38	Left visual cortex (BA17, BA18)	-32	-98	4	1 437	5.14						
		-52	-30	-8		2.86		-38	-58	-16		3.57						
	Visual cortex (BA17)	12	-92	4	281	3.86		-36	-74	-12		3.55						
							Right visual cortex (BA17, BA18), Fusiform gyrus	28	-98	0	1 260	4.78						
								22	-80	-14		3.50						
								12	-88	12		3.27						

^**1**^ Activity co-ordinates (x, y, z) are given in Montreal Neurological Institute (MNI) Atlas space.

^**2**^ Magnitude and extent statistics correspond to a minimum threshold of *P*_**FWE**_ *<* 0.05 (cluster corrected at whole-brain).

K_E_ = cluster size.

BA = Brodmann area.

#### Main analyses: between-group results

In response to acceptance, patients showed significantly decreased activation in a dorsomedial prefrontal cortex (DMPFC, Brodmann area 8-BA8-, extending to BA9) compared to controls. By contrast, patients showed increased activation of the left secondary visual cortex (parastriate BA18) during rejection (Table 2, Fig 2, and S1 Fig).

#### Interactions with clinical variables

**Fig 3:**
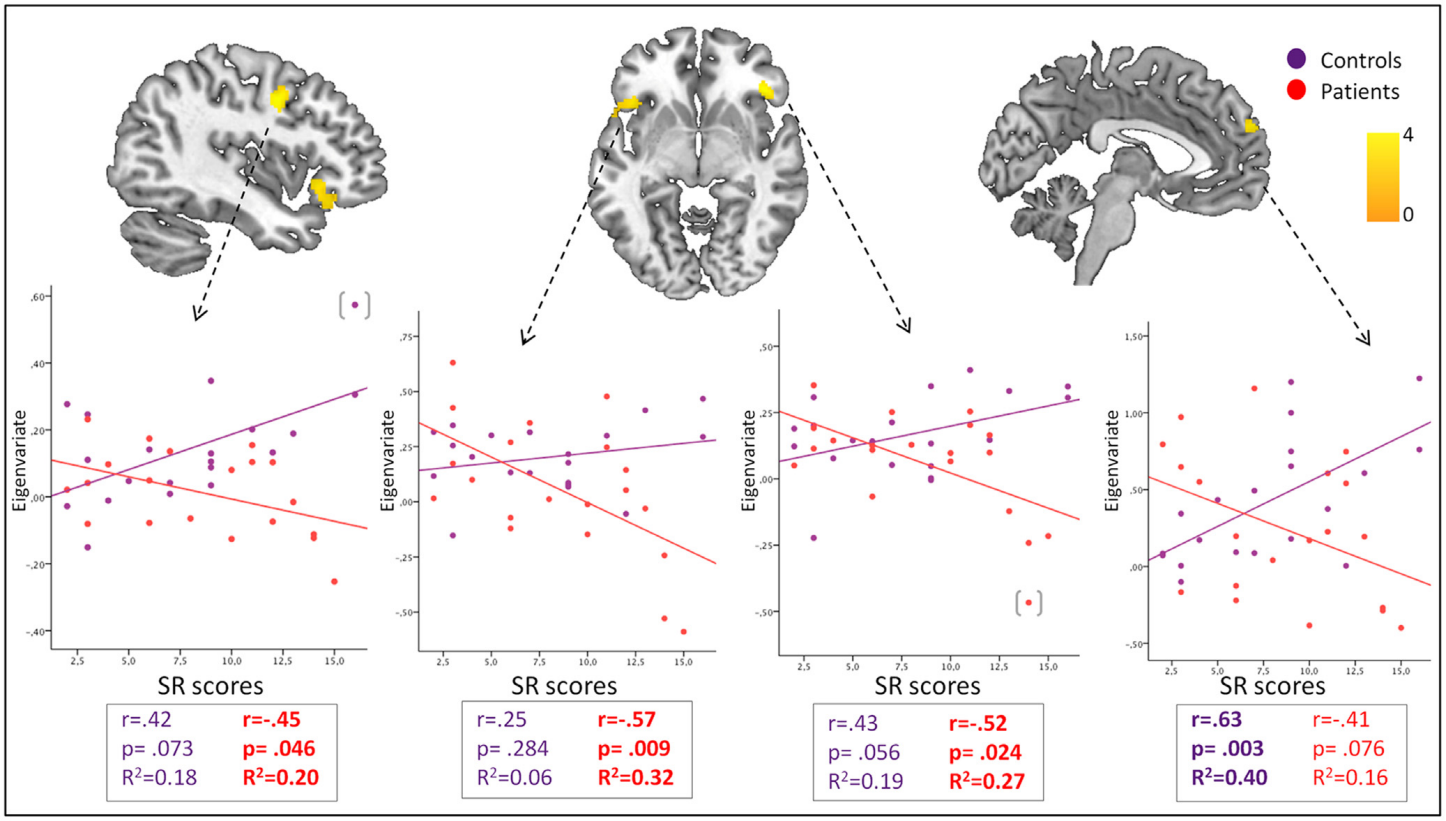
Interactions between Sensitivity to Reward and brain activations during the acceptance condition. Color bars represents T value. Images are displayed in neurological convention (left is left). Scatter plots represent Pearson's correlations between sensitivity to reward scores and the extracted mean eigenvalues in each relevant cluster: A. Dorsolateral prefrontal cortex. B. Left orbitofrontal-anterior insula cortex. C. Right orbitofrontal-anterior insula cortex. D. Dorsomedial prefrontal cortex. A results table is included, showing peak coordinates of each cluster and their corresponding statistics. (): Two outliers were detected based on the Tukey’s Outlier Filter. Although depicted in the figure, they were removed from correlation analyses.

In response to acceptance feedback, sensitivity to reward was differentially associated—between groups—with activity of bilateral frontal opercula-anterior insula cortices (negative association in patients), and the dorsomedial and dorsolateral prefrontal cortices (BA8, BA10; positive association in controls; Fig 3, S2 Table). No areas of between-group interaction were found in response to rejection feedback.

**Fig 4:**
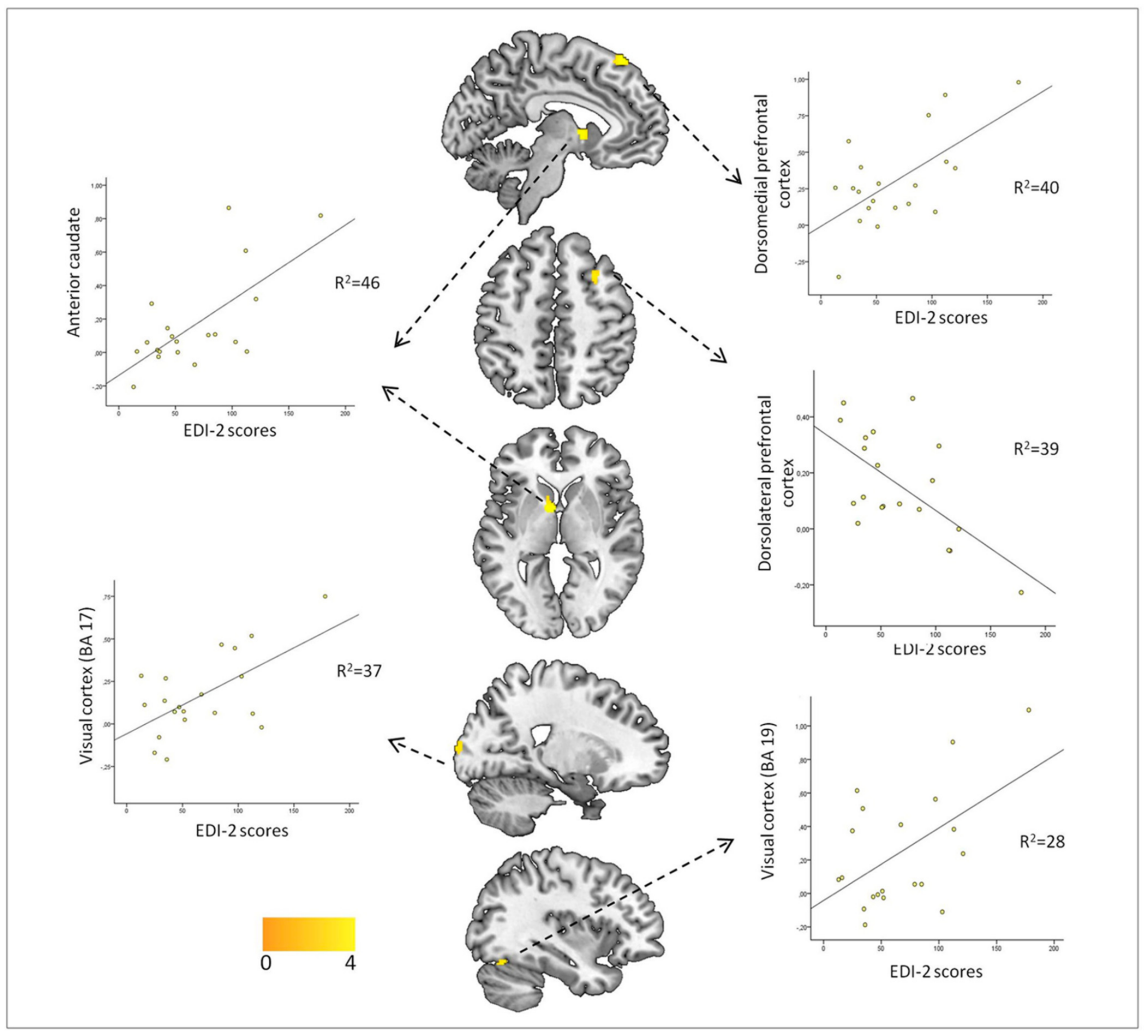
Associations between EDI-2 scores and brain activity in AN patients during rejection feedback. Color bars represents T value. Images are displayed in neurological convention (left is left). Scatter plots represent Pearson's correlations between EDI-2 scores and the extracted mean eigenvalues in each one of the significant clusters. A results table is included, showing peak coordinates of each cluster and their corresponding statistics.

There were no associations between symptom severity measured with the EDI-2 and brain activation observed in response to acceptance feedback. By comparison, symptom severity was both positively and negatively associated with brain activation observed in response to rejection feedback. Specifically, positive correlations were observed between symptom severity and ventral striatal-located at the ventral part of the caudate nucleus-, dorsomedial prefrontal (BA8), and visual cortical (BA17-BA18-BA19) activations, while negative correlations were observed with dorsolateral prefrontal cortex activation (Fig 4, S2 Table).

Several post-hoc analyses were conducted. First, and given the associations between illness duration and reward responses in other disorders [61], we extracted mean signal values from regions with significant results and correlated them with illness duration and age at onset, finding, however, no significant associations (S3 Table). Second, and because of the high prevalence of social anxiety in AN, social anxiety (LSAS) scores were included in correlation analyses using the same approach as for severity measures, to explore whether this putative contributing factor would be independently associated with brain responses to social acceptance and rejection. However, there were no associations emerging from these correlation analyses. Finally, to control for potential effects of treatment over brain activity during feedback presentations, we repeated all the above analyses excluding the 5 patients under pharmacological treatment (12.5% of the sample, 25% of the patients). Most of the results were replicated, except, for the patient group, the association between severity and brain activation during rejection at the dorsolateral prefrontal and visual cortices (BA19). Despite the lost of statistical power, the rest of results were replicated with reductions of the size of clusters with significant voxels-which therefore affected cluster-based corrected significance-, mainly at the level of bilateral anterior insula and dorsomedial prefrontal cortices in the sensitivity to reward interaction analysis (S2 Fig and legend).

## Discussion

The results of the present study suggest that alterations in reward responses to social stimuli in AN involve an overlapping network of social cognition, attentional and reward-processing areas, highlighting the tight involvement of large-scale and distributed networks in complex processes such as social feedback evaluation [62,63]. Interestingly, the activation of reward-related structures in both conditions showed paradoxical associations with either the severity of the disorder or sensitivity to reward scores, suggesting their implication in disorder-related dysfunctional processing of social reward. Alterations in brain responses to reward and punishment, which have been implicated in the pathophysiology of AN, might also relevantly contribute to the dysfunctional social relationships experienced by AN patients. Although other factors such as social anxiety symptoms might modulate responses to reward in this context, the lack of associations between brain activations to reward/punishment and LSAS scores gives further relevance to the associations found with the severity of AN.

An extensive cluster located in the dorsomedial prefrontal cortex (DMPFC), which is commonly activated by social feedback [19], was non-specifically activated in both groups and in both conditions. Nevertheless, AN patients showed hypoactivation within this region during positive feedback. The DMPFC participates in social-cognition processes such as self-reference and reflective self-knowledge [64], making inferences about how we are viewed by others [65–68], and in inhibiting the tendency of using oneself as a reference during social judgments [68]. DMPFC hypoactivations have been observed in AN patients during the performance of related tasks, such as theory of mind (attribution of intentions, BA10) [69] and self-appraisal (BA6, [70]). Additionally, the medial part of BA8 has been associated with tolerance to uncertainty [68,71,72] which, in social contexts, seems necessary for adaptively inferring other's mental state given the unpredictability of other's minds [68]. Reduced DMPFc activation in patients during acceptance suggests that self-evaluative processes and inference of other's mental states might be particularly disrupted in AN during rewarding social feedback, consistent with the reduced perception of reward value in AN [37,38], and indicating a general inhibitory-motivational response to social reward. Moreover, this response might be also associated with low tolerance to uncertainty and increased perception of lack of control in social relationships in AN patients [73], suggested to be compensated by increased control over eating, body shape and weight [10,27]. Since there was a positive association between DMPFc activity and EDI-2 scores during rejection, the opposite process—i.e increased motivational response through the engagement of the DMPFc- might be occurring when receiving negative feedback, although no between-group differences in DMPFc were found for this condition.

During rejection, the pattern of activations was more similar between groups. Activation of the attention-network (visual cortex), together with behavioral results—rejection responses were better remembered—suggests increased attention during rejection in both groups. However, AN patients presented hyperactivation of left parastriatal visual regions, which were additionally correlated with the severity of the disorder. These results are consistent with attentional biases to negative social stimuli found in behavioral studies of AN [37,74], and might again indicate a distorted motivational drive towards negative stimuli. In other disorders, such as depression and anxiety, attentional biases towards negative stimuli have been found to increase and maintain the pathological state, but also to be modifiable [75]. Attentional bias modification strategies have been suggested in AN, and might be particularly helpful in changing cognitive biases to negative social stimuli through modification of attentional pathways [75]. Consistent with previous hypotheses, our results suggest the relevance of combined alterations in social motivation and visual orienting brain areas contributing to impaired interpersonal relationships in AN, similar to what has been observed in other disorders [10,38].

We additionally observed a between-group differential pattern of associations between brain responses and sensitivity to reward. It is worth mentioning that these differences were found even though there were no differences in sensitivity to reward scores. Indeed, while it is reasonably well established that AN patients present higher sensitivity to punishment, evidences for sensitivity to reward are mixed, and, if present, they might be more relevant in samples composed by purgative rather than restrictive subtype patients [46,76]. However, these negative findings in the drive for rewards are not incompatible with alterations in either the reward perceived or in the interaction between the drive and perceived reward from social relationships. In our study, while control participants with high reward sensitivity engaged cognitive-control structures—possibly regulating an elevated motivational drive during positive feedback—AN patients showed a negative association between insula activation and sensitivity to reward. Neurobiological models of sensitivity to reward suggest its encoding in a cortico-limbic system including the reward loop linking the midbrain and ventral striatum with the prefrontal cortex [77], and, among them, the anterior insula has suggested to be more specifically involved in social rewards [78]. This region has been strongly hypothesized to be involved in the pathophysiology of AN [79], and some studies have found insula hypoactivation during the processing of primary rewarding stimuli both in ill [80] and recovered AN patients [34,81]. Indeed, these results suggest that in AN patients there is a disruption of the expected association between incentive motivation and the hedonic insular involvement in reward [82,83]. Such uncoupling of *wanting* from *liking* has been previously proposed in AN in other contexts [84] and might indicate here a dysfunctional compensatory response to a natural motivational drive for social rewards [85]. According to our results, besides its implication in altered processing of basic rewards in AN, anterior insula may also lose control over more complex reward-related responses, such as those dependent on social feedback.

Finally, the ventral striatum (VS) activation during rejection feedback was associated with the EDI-2 scores. This association was only found during rejection, and might be evidence of aberrant functioning of this system during social interactions in AN, similar to the observed VS activation when patients viewed emaciated bodies [31], or received losses in a monetary task [35]. Interestingly, the results were mainly located in the ventral part of the caudate nucleus, which has shown its involvement in reward processing particularly when feedback is involved and in the context of social learning [86]. In any case, while the above findings seem to imply that the suggested aberrant response of this structure to a range of rewards might be also mediating altered social reward-based responses, other explanations should be also taken into account. For example, VS activity might be compensating for emotional pain associated with rejection, similar to VS activation in placebo-induced analgesia [87]. The specific contribution of these factors, however, requires further investigations.

## Limitations

Our sample size was relatively modest and replication is required. However, we assessed for the first time brain responses in AN patients during social feedback, as well as their association with clinical and personality variables. Secondly, we included patients on current pharmacological treatment. Although it is unclear the direction in which treatment might bias these specific results, the exclusion of the 5 medicated participants—and despite the lost of statistical power- did not substantially modified our main results. However, medication effects cannot be ruled out, suggesting there is a need for further evaluation of this issue. Thirdly, we did not conduct a metabolic study in our protocol, which could have allowed a better characterization of the sample and the investigation of putative associations between altered metabolic variables and our findings. However, Day Unit recruitment—patients with BMI≥14 in our centre, with better metabolic profiles-, was conducted in order to minimize possible confounding effects of malnutrition on both task performance and BOLD signal. Fourthly, our study was restricted to low-weight adult AN females, with no comorbidities, and used a cross-sectional design. It will be interesting for future studies to test our results in other populations, such as patients with comorbidities, men, or adolescent samples. Moreover, although we did not find associations between our results and age at onset or illness duration, it would be equally interesting to assess the involvement of these alterations in the onset of the disorder, as well as their impact on the prognosis and outcome of patients, and whether they persist after weight restoration and symptom recovery. Longitudinal studies, the study of patients who have recovered, or intermediate phenotypes, might be of particular interest in answering these questions.

## Conclusions

Our results suggest a possible link between altered patterns of social relationships in AN and dysfunctional reward-related brain responses. These alterations might be of relevance in the maintenance of social maladaptive responses and eventually in the persistence of the disorder, and might help to explain the elevated resistance to change in patients with AN. Although alterations to functioning of the reward system have been highlighted recently in several psychiatric disorders, given the rewarding nature of food and the involvement of the reward circuit in food consumption (i.e. insula and frontal operculum, ventral striatum and amygdala, midbrain and frontal cortex) [88], these associations are of particular relevance for eating disorders such as AN [27]. In view of our findings it would be interesting for future studies to test the effectiveness of reward-processing-focused treatments, which might be easily included in therapies such as cognitive remediation or fMRI-based neurofeedback training. For example, patients with anorexia nervosa might be trained to engage specific structures (e.g. DMPFc, ventral striatum) in front of social rewarding contexts such as social approval [89], which might ultimately improve their social responses and functional impairment. However, to our knowledge, there have been no studies using neurofeedback in AN, and the use of neurofeedback with complex stimuli such as social responses is still a field in development [90]. Additionally, it would be also of interest to examine other aspects of reward processing in social settings, such as the influence of reward expectations and prediction error in social relationships. Similar paradigms might also be interesting in the context of current trials on oxytocin [91], to evaluate treatment-mediated changes in the processing of social stimuli in AN.

## Supporting Information

S1 Fig Parameter estimates (β values) of the main conditions. Footnote: Bar charts represent parameter estimates at the medial prefrontal cortex (x,y,z = 10,32,50) and visual cortex-BA18 (x,y,z = -30, -98, 6).
(DOC)

S2 Fig Overlapping maps of between-group differences including and excluding patients on pharmacological treatment. Footnote: [A] Main task comparisons: Acceptance: vmPFC: 68voxels, Z = 3.01, PFWE-equivalent = .04; Rejection: visual cortex (paraestriate BA18): 31voxels, Z = 2.90, PFWE-equivalent = .05. [B] Correlations: Acceptance-sensitivity to reward interaction: dorsolateral prefrontal cortex: 61voxels, Z = 3.17, PFWE-equivalent = .04; DMPFc: 5 voxels, Z = 2.92, PFWE-equivalent>.05; right frontal opercula-insula: 9 voxels, Z = 2.69, PFWE-equivalent>.05; left frontal opercula-insula: 1 voxel, Z = 2.59, PFWE-equivalent>.05. Rejection-severity correlation: DMPFc: 82 voxels, Z = 3.30, PFWE-equivalent = .01; anterior caudate (ventral striatum): 7voxels, Z = 2.69, PFWE-equivalent = .05; visual cortex (BA17): 10voxels, Z = 2.87, PFWE-equivalent = .05) (Dorsolateral prefrontal cortex and visual cortex (BA19) were not present even when lowering the voxel threshold to .01). PFWE-equivalent indicates the Alphasim cluster-based corrected significance, with a minimum threshold of p>.005 uncorrected at the voxel-level.
(DOC)

S1 Table Behavioral responses of the subjects included in the study. Footnote: *Recall of rejection>recall of acceptance>recall of no feedback, all p <.001. ‡ Scores to faces giving rejection> scores to faces giving acceptance; Scores to faces giving rejection> scores to faces giving no feedback, both for pre and post scores and all p <.001. † Accepted>no feedback given>rejected, all p <.001.
(DOC)

S2 Table Coordinates and statistics of correlation and interaction analyses. (DOC)

S3 Table Correlations between extracted eigenvalues of significant regions and clinical variables of interest. (DOC)
