# Supplementary material for: Abnormal Social Reward Responses in Anorexia Nervosa: An fMRI Study
Source: PLoS One. 2015 Jul 21;10(7):e0133539. doi: 10.1371/journal.pone.0133539 (PMC4510264; doi:10.1371/journal.pone.0133539)
Supplement: S1 Fig — Footnote: Bar charts represent parameter estimates at the medial prefrontal cortex (x,y,z = 10,32,50) and visual cortex-BA18 (x,y,z = -30, -98, 6). (DOC) [file pone.0133539.s001.doc]

**S1 Fig. Parameter estimates (β values) of the main conditions**.

**
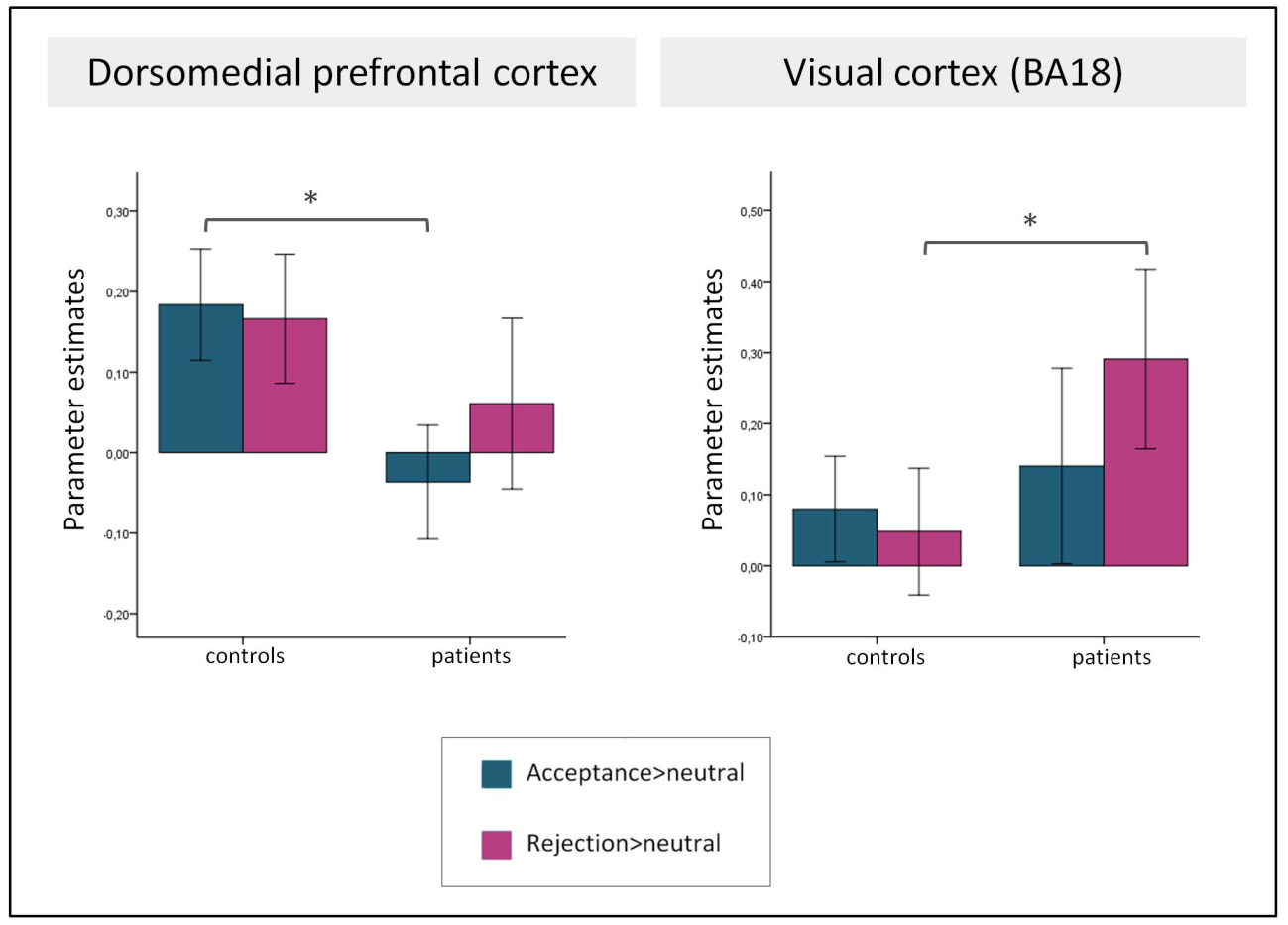
**

Bar charts represent parameter estimates at the medial prefrontal cortex (x,y,z=10,32,50) and visual cortex-BA18 (x,y,z=-30,-98, 6).
