# Supplementary material for: Abnormal Social Reward Responses in Anorexia Nervosa: An fMRI Study
Source: PLoS One. 2015 Jul 21;10(7):e0133539. doi: 10.1371/journal.pone.0133539 (PMC4510264; doi:10.1371/journal.pone.0133539)
Supplement: S2 Fig — Footnote: [A] Main task comparisons: Acceptance: vmPFC: 68voxels, Z = 3.01, PFWE-equivalent = .04; Rejection: visual cortex (paraestriate BA18): 31voxels, Z = 2.90, PFWE-equivalent = .05. [B] Correlations: Acceptance-sensitivity to reward interaction: dorsolateral prefrontal cortex: 61voxels, Z = 3.17, PFWE-equivalent = .04; DMPFc: 5 voxels, Z = 2.92, PFWE-equivalent>.05; right frontal opercula-insula: 9 voxels, Z = 2.69, PFWE-equivalent>.05; left frontal opercula-insula: 1 voxel, Z = 2.59, PFWE-equivalent>.05. Rejection-severity correlation: DMPFc: 82 voxels, Z = 3.30, PFWE-equivalent = .01; anterior caudate (ventral striatum): 7voxels, Z = 2.69, PFWE-equivalent = .05; visual cortex (BA17): 10voxels, Z = 2.87, PFWE-equivalent = .05) (Dorsolateral prefrontal cortex and visual cortex (BA19) were not present even when lowering the voxel threshold to .01). PFWE-equivalent indicates the Alphasim cluster-based corrected significance, with a minimum threshold of p>.005 uncorrected at the voxel-level. (DOC) [file pone.0133539.s002.doc]

**S2 Fig. Overlapping maps of between-group differences including and excluding patients on pharmacological treatment.**

**
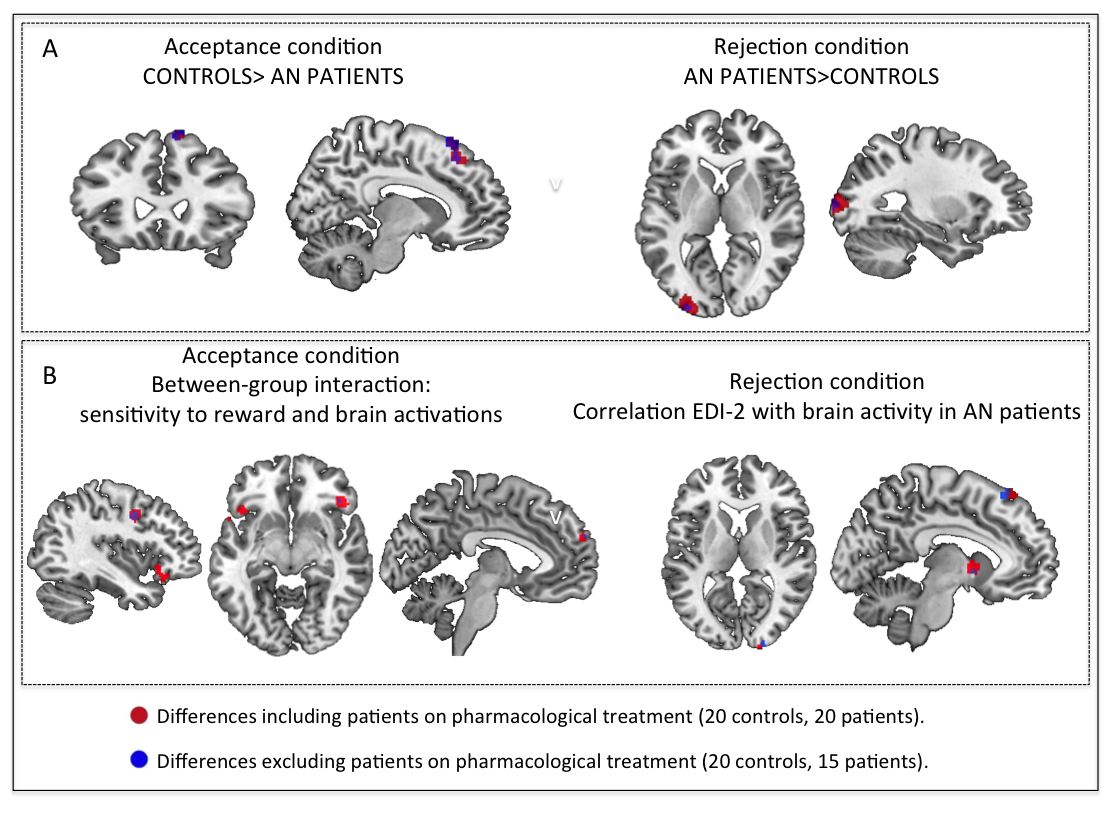
**

[A] Main task comparisons: **Acceptance:** vmPFC: 68voxels, Z= 3.01, P_FWE_-equivalent= .04; **Rejection**: visual cortex (paraestriate BA18): 31voxels, Z= 2.90, P_FWE_-equivalent= .05. [B] Correlations: **Acceptance-sensitivity to reward interaction**: dorsolateral prefrontal cortex: 61voxels, Z=3.17, P_FWE_-equivalent=.04; DMPFc: 5 voxels, Z= 2.92, P_FWE_-equivalent>.05; right frontal opercula-insula: 9 voxels, Z=2.69, P_FWE_-equivalent>.05; left frontal opercula-insula: 1 voxel, Z=2.59, P_FWE_-equivalent>.05. **Rejection-severity correlation**: DMPFc: 82 voxels, Z=3.30, P_FWE_-equivalent=.01; anterior caudate (ventral striatum): 7voxels, Z=2.69, P_FWE_-equivalent=.05; visual cortex (BA17): 10voxels, Z=2.87 , P_FWE_-equivalent=.05) (Dorsolateral prefrontal cortex and visual cortex (BA19) were not present even when lowering the voxel threshold to .01).

P_FWE_-equivalent indicates the Alphasim cluster-based corrected significance, with a minimum threshold of *p*>.005 uncorrected at the voxel-level.
