# Supplementary material for: Abnormal Social Reward Responses in Anorexia Nervosa: An fMRI Study
Source: PLoS One. 2015 Jul 21;10(7):e0133539. doi: 10.1371/journal.pone.0133539 (PMC4510264; doi:10.1371/journal.pone.0133539)
Supplement: S1 Table — Footnote: *Recall of rejection>recall of acceptance>recall of no feedback, all p <.001. ‡ Scores to faces giving rejection> scores to faces giving acceptance; Scores to faces giving rejection> scores to faces giving no feedback, both for pre and post scores and all p <.001. † Accepted>no feedback given>rejected, all p <.001. (DOC) [file pone.0133539.s003.doc]

**S1 Table. Behavioral responses of the subjects included in the study**.

| **Behavioural-task related measurements, mean (SD)** |  | **AN patients (n=20)** | **Healthy controls (n=20)** | **t/F statistics** | **p** | ***Partial eta squared*** |
| --- | --- | --- | --- | --- | --- | --- |
|  |  |  |  |  |  |  |
| Number of correctly classified faces (appearing or not during the fMRI task) (out of 70 faces) |  | 48.20 (7.62) (69%) | 45.79 (8.80) (65%) | t(37)=0.92 | .37 | .02 |
|  |  |  |  |  |  |  |
| Accuracy in the recall of feedback responses (out of 54 faces) |  | 18.60 (7.18) (34%) | 18.80 (8.48) (35%) |  |  |  |
|  | Recall of acceptance responses (out of 17) | 6.05 (2.50) | 5.75 (2.77) |  |  |  |
|  | Recall of rejection responses (out of 18) | 7.75 (2.77) | 7.40 (3.25) |  |  |  |
|  | Recall of no feedback responses (out of 19) | 4.80 (2.78) | 5.65 (3.92) |  |  |  |
|  | Group effects |  |  | F(1,38)=0.01 | .94 | ~0 |
|  |  |  |  |  |  |  |
|  | Condition effects |  |  | F(2,76)=16.54 | <.001* | .30 |
|  |  |  |  |  |  |  |
|  | Interaction effects |  |  | F(2,76)=1.30 | .28 | .03 |
|  |  |  |  |  |  |  |
| Scores given to face database (pre and post-scanning) |  |  |  |  |  |  |
| (0-10) |  |  |  |  |  |  |
|  | To faces giving acceptance -pre | 5.06 (0.65) | 5.29 (0.02) |  |  |  |
|  | To faces giving acceptance -post | 5.17 (0.71) | 5.19 (0.07) |  |  |  |
|  | To faces giving rejection -pre | 5.44 (0.05) | 5.59 (0.54) |  |  |  |
|  | To faces giving rejection -post | 5.41 (0.72) | 5.44 (0.70) |  |  |  |
|  | To faces giving no feedback –pre | 5.02 (0.78) | 5.11 (0.53) |  |  |  |
|  | To faces giving no feedback –post | 5.07 (0.71) | 5.11 (0.56) |  |  |  |
|  |  |  |  |  |  |  |
|  | Group effects |  |  | F(1,38)=0.29 | .59 | .01 |
|  |  |  |  |  |  |  |
|  | Condition effects |  |  | F(2,76)=36.43 | <.001‡ | .49 |
|  |  |  |  |  |  |  |
|  | Scores by condition effects |  |  | F(2,76)=0.51 | .54 | .01 |
|  |  |  |  |  |  |  |
|  | Interaction (scores by condition by group) effects |  |  | F(2,76)=0.27 | .69 | .01 |
|  |  |  |  |  |  |  |
| Scores of “how did you feel when being…” (0-10) |  | (N=19) | (N=20) |  |  |  |
|  | Accepted | 8.05 (1.54) | 7.63 (1.05) |  |  |  |
|  | Rejected | 4.05 (1.81) | 3.65 (1.35) |  |  |  |
|  | No feedback given | 4.90 (0.46) | 5.16 (1.09) |  |  |  |
|  | Group effects |  |  | F(1,37) =0.99 | .32 | .03 |
|  |  |  |  |  |  |  |
|  | Condition effects |  |  | F(2,74) =83.32 | <.001† | .69 |
|  |  |  |  |  |  |  |
|  | Interaction effects |  |  | F(2,74) =0.77 | .43 | .02 |
|  |  |  |  |  |  |  |
| Believe of being rated (0-10) |  | (N=15) | (N=16) |  |  |  |
|  |  | 9.4 (1.30) | 9.12 (1.21) | t(29)=0.62 | .54 | .01 |

*Recall of rejection>recall of acceptance>recall of no feedback, all p<.001. ‡ Scores to faces giving rejection> scores to faces giving acceptance; Scores to faces giving rejection> scores to faces giving no feedback, both for pre and post MRI scores and all p<.001. † Accepted>no feedback given>rejected, all p<.001.
