# Supplementary material for: Abnormal Social Reward Responses in Anorexia Nervosa: An fMRI Study
Source: PLoS One. 2015 Jul 21;10(7):e0133539. doi: 10.1371/journal.pone.0133539 (PMC4510264; doi:10.1371/journal.pone.0133539)
Supplement: S2 Table — (DOC) [file pone.0133539.s004.doc]

**S2 Table. Coordinates and statistics of correlation and interaction analyses**.

| **Correlation in AN patients between EDI-2 scores and brain activation in response to the rejection condition** | | | | | |
| --- | --- | --- | --- | --- | --- |
| **Anatomy** | **Coordinates** | | | **Stats** | |
| Dorsomedial prefrontal cortex (BA 8) | -8 | 38 | 60 | 71 | 3.70 |
| Dorsolateral prefrontal cortex (BA 8) | 30 | 14 | 50 | 40 | 2.97 |
| Anterior caudate | -10 | 4 | 0 | 67 | 3.70 |
|  | -10 | 14 | 0 |  | 2.75 |
| Associative visual cortex (BA 19) | -34 | -68 | -20 | 22 | 3.18 |
| Primary visual cortex (BA 17 extending to BA18) | 18 | -100 | 10 | 16 | 3.00 |
| **Interactions between Sensitivity to Reward and brain activations in AN patients and controls in response to the acceptance condition.** | | | | | |
| **Anatomy** | **Coordinates** | | | **Stats** | |
|  | **x** | **y** | **z** | K_E_ | **Z** |
| Right frontal opercula-anterior insula complex / Inferior frontal-lateral orbitofrontal cortex | 44 | 30 | -10 | 144 | 3.76 |
| Left frontal opercula-anterior insula complex /Inferior frontal cortex-anterior insula | -56 | 12 | 12 | 312 | 3.12 |
|  | -42 | 22 | -6 |  | 3.11 |
|  | -44 | 30 | -16 |  | 3.08 |
| Dorsolateral prefrontal cortex (BA6) | -42 | 0 | 40 | 120 | 3.67 |
| Dorsomedial prefrontal cortex (BA10) | -6 | 62 | 28 | 120 | 3.49 |
|  | 6 | 60 | 26 |  | 3.03 |
