# Supplementary material for: Abnormal Social Reward Responses in Anorexia Nervosa: An fMRI Study
Source: PLoS One. 2015 Jul 21;10(7):e0133539. doi: 10.1371/journal.pone.0133539 (PMC4510264; doi:10.1371/journal.pone.0133539)
Supplement: S3 Table — (DOC) [file pone.0133539.s005.doc]

|  |  | Illness duration (months) | Age at the onset |
| --- | --- | --- | --- |
| Acceptance condition | Dorsomedial prefrontal cortex | r=.10 *p*=.68 | r=.19 *p*=.42 |
|  | Right frontal opercula- anterior insula | r=-.09, *p*=.72 | r=.31, *p*=.19 |
|  | Left frontal opercula-anterior insula | r=-.01, *p*=.95 | r=.30, *p*=.19 |
|  | Dorsolateral prefrontal cortex | r=-.15 *p*=.52 | r=.39 *p*=.09 |
| Rejection condition | Visual cortex (BA18) | r=-.20 *p*=.39 | r=.18 *p*=.46 |
|  | Ventral striatum (anterior caudate) | r=-.36 *p*=.15 | r=-.45, *p*=.07 |
|  | Dorsomedial prefrontal cortex | r= -.14 *p*=.56 | r=.03 *p*=.91 |
|  | Dorsolateral prefrontal cortex | r=.18 *p*=.46 | r=-.22 *p*=.35 |
|  | Visual cortex (BA19) | r=-.004 *p*=.99 | r=-.18 *p*=.44 |
|  | Visual cortex (BA17) | r= -.41 *p*=.08 | r=-.12 *p*=.63 |

**S3 Table. Correlations between extracted eigenvalues of significant regions and clinical variables of interest.**
